# Interleukin-18 Expression Increases in the Aorta and Plasma of Patients with Acute Aortic Dissection

**DOI:** 10.1155/2019/8691294

**Published:** 2019-07-22

**Authors:** Haiying Hu, Guangtai Zhang, Huanli Hu, Wenjing Liu, Jixiang Liu, Shuanli Xin, Xiufeng Zhao, Liying Han, Liping Duan, Xinshun Huang, Chao Chang

**Affiliations:** ^1^Department of Cardiology, Handan First Hospital, Handan 056002, China; ^2^Department of Geriatrics, Handan Central Hospital, Handan 056002, China; ^3^Department of Cardiology, The People's Hospital of Langfang City, Langfang 065000, China; ^4^Department of Cardiology, The People's Hospital of Guangxi Zhuang Autonomous Region, Nanning 530021, China

## Abstract

**Background:**

Interleukin- (IL-) 18 is a proinflammatory cytokine related to cardiovascular diseases, including hypertension and atherosclerosis. This study is aimed at determining whether IL-18 is related to aortic dissection (AD) and identifying the underlying mechanisms.

**Methods:**

IL-18 expression in human aorta samples from AD (*n* = 8) and non-AD (NAD, *n* = 7) patients was measured. In addition, the IL-18, IL-6, interferon- (IFN-) *γ*, and IL-18-binding protein (IL-18BP) concentrations in plasma samples collected from the NAD and AD patients were detected. The effects of IL-18 on macrophage differentiation and smooth muscle cell (SMC) apoptosis were investigated in vitro.

**Results:**

IL-18 expression was significantly increased in the aorta samples from the AD patients compared with those from the NAD patients, especially in the torn section. Aortic IL-18 was mainly derived from macrophages and also partly derived from CD4+ T lymphocytes and vascular SMCs. Plasma IL-18, IFN-*γ*, and IL-6 levels were significantly higher in the AD group than in the NAD group, and the IL-18 levels were positively correlated with the IFN-*γ* and IL-6 levels. In addition, plasma IL-18BP and free IL-18 levels were also elevated in the AD group. Linear regression analysis showed that the IL-18 level was independently associated with the presence of AD. In addition, anti-mouse IL-18-neutralizing monoclonal antibodies (anti-IL-18 nAb) inhibited angiotensin II-induced M1 macrophage differentiation and SMC apoptosis in vitro.

**Conclusion:**

IL-18 may participate in AD by regulating macrophage differentiation and macrophage-induced SMC apoptosis.

## 1. Introduction

Given its features of acute onset, rapid development, and high morbidity and mortality, acute aortic dissection (AD) is a severe cardiovascular disease. A previous study reported that the hourly mortality rate of early acute AD is approximately 1%-2% after the onset of symptoms [1]. However, common diagnostic methods, such as electrocardiography, chest radiography, and computed tomography (CT), are either unavailable at the bedside or time-consuming, and the misdiagnosis rate may be approximately 38% [2]. Although the specific mechanism of AD remains unknown, studies have demonstrated that the inflammatory response is associated with AD [3, 4].

Interleukin- (IL-) 18 belongs to the IL-1 family and was originally identified as an interferon- (IFN-) *γ*-inducing factor [5]. The main sources of IL-18 in humans are immune cells, such as monocytes, macrophages, dendritic cells, and B lymphocytes [6]. IL-18 has been reported to be involved in many inflammatory and immune diseases by promoting the expression of other proinflammatory cytokines. IL-18 can promote IFN-*γ* secretion, which is one of the most important mechanisms of type 1 diabetes (T1D) [7]. In addition, IL-18 expression is also increased in human atopic eczema, possibly via the upregulation of the T helper 1 (Th1) immune response and downregulation of the T helper 2 (Th2) immune response, which aggravates the imbalance between Th1 and Th2 cells and results in the deterioration etiology of atopic eczema seen in humans [8, 9]. However, the biological actions of IL-18 can be countered by its endogenous antagonist, termed IL-18-binding protein (IL-18BP) [10].

IL-18 has also been reported to be closely related to a variety of cardiovascular diseases. An earlier study reported that IL-18 levels are a strong independent factor of death from cardiovascular causes in patients with coronary artery disease [11]. In a recent study, a change in IL-18 levels was found to be useful in the diagnosis of acute coronary syndrome (ACS) and prediction of its prognosis [12]. In addition, circulating IL-18 levels are elevated in patients with atrial fibrillation and may be superior to other inflammatory markers that are known to have elevated levels [13]. However, the IL-18 expression pattern in AD remains unknown, and this study is aimed at detecting IL-18 expression in human AD and exploring possible mechanisms underlying the participation of IL-18 in AD.

## 2. Materials and Methods

### 2.1. Collection of Human Aortic Samples and Blood Samples

A total of 8 aortic samples were obtained from patients who suffered from thoracic aortic dissection (TAD) and received aortic replacement surgery; 7 control aortic samples were collected from organ donors who suffered accidental death and did not exhibit obvious cardiovascular disease. From January 2017 to May 2018, consecutive patients (*n* = 150) who were hospitalized and received computed tomography angiography (CTA) after experiencing sudden chest pain were included in this study. Patients who had a history of autoimmune disease (including rheumatoid arthritis, *n* = 2; psoriasis, *n* = 1; and systemic lupus erythematosus, *n* = 3) or cardiovascular disease (including coronary artery disease, *n* = 11; valvular heart disease, *n* = 5; viral myocarditis, *n* = 3; pulmonary arterial hypertension (PAH), *n* = 2; and heart failure, *n* = 4) were excluded. The remaining 119 patients were divided into a non-AD group (NAD group, *n* = 31) and an AD group (*n* = 88) according to their clinical symptoms and CTA results. The AD group was further divided into a Stanford A group (45 patients) and Stanford B group (43 patients).

All aortic samples and blood samples were collected at the People's Hospital of Guangxi Zhuang Autonomous Region as described in our previous studies [14, 15]. This study was approved by the Medical Ethics Committee of the People's Hospital of Guangxi Zhuang Autonomous Region and the Handan First Hospital, and the patients or their families provided informed consent.

### 2.2. Western Blot Analysis

Total protein was successfully extracted from aortic tissue samples in RIPA lysis buffer. Protein concentrations were detected and quantified using the BCA Protein Assay Kit (Thermo Fisher Scientific, USA). A total of 20 *μ*g of total protein was separated by sodium dodecyl sulfate-polyacrylamide gel electrophoresis. The proteins were transferred to a polyvinylidene fluoride membrane. The membranes were blocked with 5% nonfat milk and then incubated with an anti-IL-18 antibody (Abcam, UK) and anti-GAPDH antibody (Cell Signaling Technology, USA) overnight at 4°C, followed by incubation with a secondary antibody at room temperature for 1 hour. The blots were detected using a two-color infrared imaging system (Odyssey, LICOR) to quantify protein expression.

### 2.3. Real-Time Quantitative PCR (qRT-PCR)

Total RNA was extracted from thoracic aortic tissue samples and cells using TRIzol reagent, and cDNA was synthesized from 2 *μ*g of total mRNA using oligo (dT) primers and a reverse transcription kit according to the manufacturer's instructions. PCR amplifications were performed using LightCycler 480 SYBR Green Master Mix (all from Roche). The sequences of the primers are listed in Table 1. Relative mRNA expression levels were measured and normalized to the GAPDH expression level in the corresponding sample.

### 2.4. Immunofluorescence

Aortic samples were fixed with 4% neutral paraformaldehyde, embedded in paraffin, cut into 4 to 5 mm sections, and mounted onto slides. Immunofluorescence staining was used to detect the expression of IL-18 in each sample. In addition, double immunofluorescence staining using an anti-CD4 antibody and anti-IL-18 antibody, anti-CD68 antibody and anti-IL-18 antibody, or anti-*α*-SMA antibody and anti-IL-18 antibody was performed.

### 2.5. Measurement of Cytokine Levels

All samples were thawed at 4°C, and plasma IL-18, IFN-*γ*, IL-6, and IL-18BP (triple from eBioscience) levels were measured using commercially available enzyme-linked immune sorbent assay (ELISA) kits according to the manufacturer's instructions. The amount of free IL-18 was calculated based on the mass action law using a dissociation constant of 400 pM and a stoichiometric ratio of 1 : 1 [16].

### 2.6. Isolation of Macrophages and CD4+ T Lymphocytes

Male wild-type (WT) mice (HFK Bioscience, Beijing, China) on the C57BL/6 background and aged 10-12 weeks were used in this study. Bone marrow-derived macrophages (Møs) were prepared as described previously [17, 18]. The isolated macrophages were plated in complete DMEM supplemented with murine macrophage colony-stimulating factor (50 ng/ml) and cultured to allow macrophage differentiation. Spleens isolated from WT mice were mashed, and the splenocyte suspension was purified using a CD4+ cell isolation kit (Miltenyi Biotec, Auburn, CA). Then, the isolated T lymphocytes (TCs) were cultivated in complete RPMI 1640 medium and activated by treatment with anti-CD3 and anti-CD28 antibodies.

### 2.7. Cell Culture Experiments

Macrophages and CD4+ TCs isolated as described above were divided into the following groups: (1) macrophages, (2) macrophages+Ang II (100 nmol/l, Sigma), (3) macrophages+CD4+ TCs+AngII, and (4) macrophages+CD4+ TCs+Ang II+anti-mouse IL-18-neutralizing monoclonal antibody (anti-IL-18 nAb; 100 ng/ml, R&D Systems). The numbers of macrophages and CD4+ TCs in each group were 5 × 10^5^ and 2.5 × 10^6^, respectively. After treatment for 24 hours in complete DMEM, the iNOS, IFN-*γ*, and IL-6 mRNA levels in the macrophages were detected by qRT-PCR. In addition, SMCs were treated with the supernatant of the cultures described above and Ang II for 12 hours, and the Bax and Bcl2 mRNA levels in the SMCs were measured.

### 2.8. Statistical Analysis

We first analyzed whether the cytokine and clinical characteristic data conformed to a normal distribution. Data with a normal distribution were expressed as the mean ± standard deviation (SD), and differences between two groups were assessed using Student's *t*-tests, while differences between more than two groups were compared by one-way analysis of variance (ANOVA) followed by Tukey's multiple comparison test. Data with a nonnormal distribution were expressed as the median (lower quartile to upper quartile) and were compared by the Mann–Whitney *U* test. Categorical variables are presented as counts (percentages) and were compared with the chi-square test. Spearman's correlation analysis was used to calculate correlations among plasma IL-18, IFN-*γ*, IL-6, and clinical characteristics. To identify independent predictors of the onset of AD, univariate and multivariate linear regression analyses were performed. A value of *P* < 0.05 was considered to indicate statistical significance. Statistical analysis was performed using GraphPad Prism7.

## 3. Results

### 3.1. Baseline Clinical Characteristics of the Patients Who Provided Aortic Tissue Samples

There were no significant differences in age, gender, uncontrolled blood pressure (HBP), smoking, the fasting glucose (Glu) level, systolic blood pressure (SBP), diastolic blood pressure (DBP), the total cholesterol (TC) level, the total triglyceride (TG) level, the high-density lipoprotein cholesterol (HDL-C) level, the low-density lipoprotein cholesterol (LDL-C) level, the creatinine (CREA) level, or heart rate (HR) between the TAD group and the control group. In contrast, the C-reaction protein (CRP) level, white blood cell (WBC) count, and D-dimer level were significantly increased in the TAD group compared with the control group. The clinical characteristics of these patients are shown in Table 2.

### 3.2. Expression and Source of IL-18 in Human Thoracic Aortic Tissue

**Figure 1:**
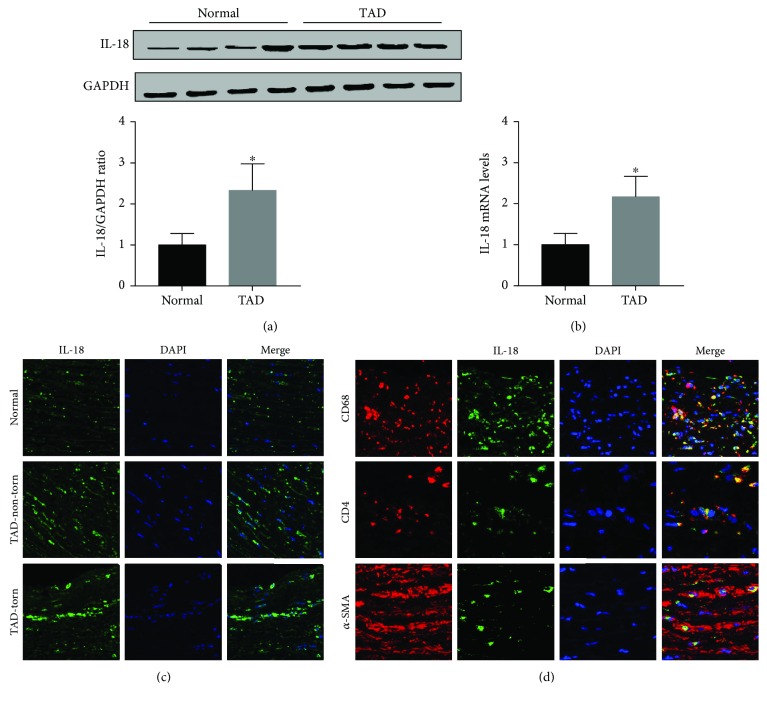
The IL-18 expression in human thoracic aortic tissues. (a) Aortic IL-18 protein levels in normal and TAD aorta tissues were detected by Western blot. (b) Aortic IL-18 mRNA levels in these two groups were measured by RT-PCR. (c) Immunofluorescence analysis of IL-18 expression in human thoracic aortic tissues (200x). (d) Double immunofluorescence staining analysis of IL-18 source in TAD aortas (400x). ^∗^*P* < 0.05 vs. the normal group.

IL-18 expression in human AD aortic samples was first detected by Western blot analysis and RT-qPCR. The results showed that aortic IL-18 levels were significantly increased in the TAD patients compared with the control group (Figures 1(a) and 1(b)). In addition, the results of immunofluorescence staining showed that higher IL-18 levels were observed in the samples of aorta, especially in the torn region, from the TAD patients (Figure 1(c)). In addition, double staining was performed to detect the main source of IL-18. The results showed that IL-18 was mainly expressed in macrophages, although small amounts were also expressed in T lymphocytes and vascular smooth muscle cells (SMCs) in human thoracic aortic tissue (Figure 1(d)).

### 3.3. Basic Clinical Characteristics of the Patients Who Provided Blood Samples

There were no significant differences in age, HBP, SBP, DBP, HR, medications, or the levels of TC, TG, HDL-C, LDL-C, or CRP between the NAD group and AD group. The incidence rate of smoking and the proportion of males as well as the Glu level, WBC count, and D-dimer level were significantly increased in the AD group compared with the NAD group. However, there were no differences between the Stanford A group and the Stanford B group in the clinical characteristics. The clinical characteristics of each group are listed in Table 3.

### 3.4. Plasma Cytokine Concentrations in the AD Patients

**Figure 2:**
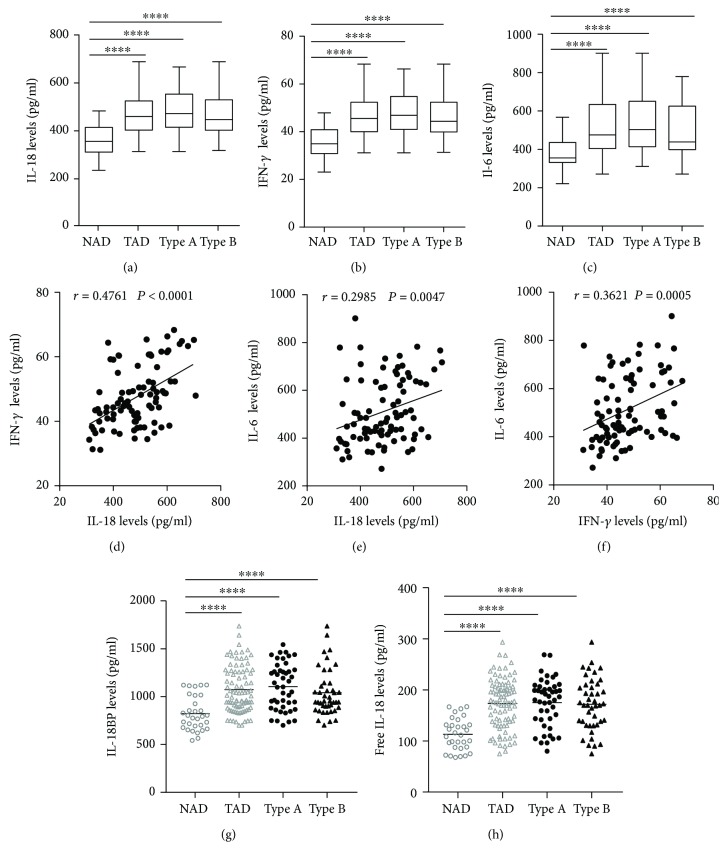
Plasma cytokine levels in NAD and AAD groups. Plasma IL-18 (a), IFN-*γ* (b), and IL-6 (c) concentrations in NAD, AAD, Stanford A, and Stanford B groups. The correlation between IL-18 (d), IL-6 (e), and IFN-*γ* levels (f) in AAD patients. Plasma IL-18BP levels (g) and the free IL-18 (h) in NAD, AAD, Stanford A, and Stanford B groups. ^∗∗∗∗^*P* < 0.0001 vs. the NAD group.

The plasma levels of IL-18, IFN-*γ*, and IL-6 were detected. The results showed that compared with the NAD group, the AD group had higher IL-18, IFN-*γ*, and IL-6 levels (Figures 2(a)–2(c)), whereas no significant differences in these three cytokines were found between the Stanford A group and the Stanford B group (Figures 2(a)–2(c)). The plasma levels in each group are shown in Table 4. Furthermore, the plasma IL-18 levels were positively correlated with the IFN-*γ* levels (*r* = 0.4761, *P* < 0.001) and the IL-6 levels (*r* = 0.2985, *P* < 0.01) in the AD patients (Figures 2(d) and 2(e)). The IFN-*γ* levels were also positively correlated with the IL-6 levels (*r* = 0.3621, *P* < 0.01) (Figure 2(f)). In addition, we detected the IL-18BP levels in the plasma. The results are listed in Table 4. The results showed that the IL-18BP level and calculated free IL-18 level were also elevated in the AD group compared with the NAD group (Figures 2(g) and 2(h)).

### 3.5. Univariate and Multivariate Linear Regression Analyses

To determine whether IL-18 is an independent predictor of the presence of AD, univariate and multivariate linear regression analyses were performed. The univariate analysis showed that the IL-18, IFN-*γ*, IL-6, Glu, CREA, and D-dimer levels; gender; and smoking status exhibited a trend towards an association with the presence of AD, whereas SBP, DBP, the TG level, the TC level, the CRP level, the WBC count, age, and HR showed no obvious association. Furthermore, the IL-18, IFN-*γ*, IL-6, Glu, CREA, and D-dimer levels; gender; and smoking status were used to perform multivariate linear regression analysis, and the results showed that the IL-18 level was independently associated with the presence of AD (*β* = 0.238, 95% confidence interval (CI) 0.053 to 0.422; *P* = 0.012). In addition, the IFN-*γ* (*β* = 0.232, 95% CI 0.044 to 0.419; *P* = 0.016), IL-6 (*β* = 0.163, 95% CI 0.002 to 0.324; *P* = 0.048), and D-dimer (*β* = 0.179, 95% CI 0.014 to 0.343; *P* = 0.034) levels were also associated with the presence of AD (as shown in Table 5).

### 3.6. Effect of IL-18 on Macrophage Differentiation and SMC Apoptosis

**Figure 3:**
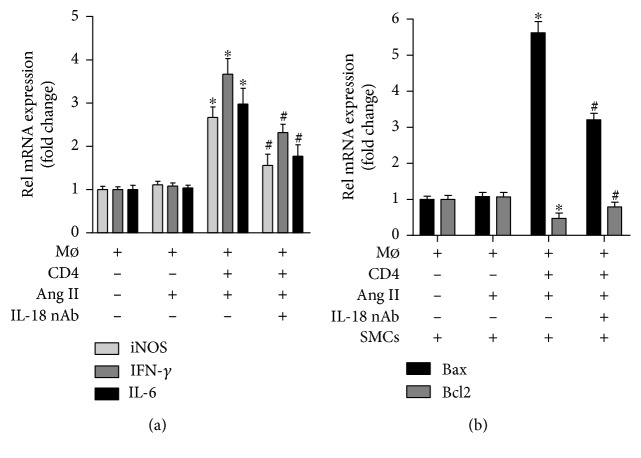
Effect of IL-18 nAb on macrophage differentiation and SMC apoptosis. (a) iNOS, IFN-*γ*, and IL-6 mRNA levels in macrophage were measured by RT-qPCR. ^∗^*P* < 0.05 vs. the Mø group; ^#^*P* < 0.05 vs. the Mø+CD4+TCs+Ang II group. (b) The Bax and Bcl2 mRNA levels in SMCs were measured by RT-qPCR. ^∗^*P* < 0.05 vs. the Mø+SMC group; ^#^*P* < 0.05 vs. the Mø+CD4+ TCs+Ang II+SMC group.

The results of cell culture experiments showed that Ang II treatment alone did not affect iNOS expression, but the iNOS level exhibited a significant increase after CD4+ T lymphocytes were added, and the iNOS level increase was partly reversed by the anti-IL-18 nAb (Figure 3(a)). A similar trend was observed for the IFN-*γ* and IL-6 mRNA levels (Figure 3(a)). In addition, the medium from the macrophage group and macrophage+Ang II group did not affect Bax and Bcl2 mRNA expression in SMCs; the Bax mRNA levels were increased, while the Bcl2 mRNA levels were decreased after treatment with the medium from the macrophage+Ang II+CD4+ T lymphocyte group, and these effects on Bax and Bcl2 expression were also reversed by the anti-IL-18 nAb (Figure 3(b)).

## 4. Discussion

In this study, we found that the level of the proinflammatory cytokine IL-18 was significantly increased in AD aortic tissue and demonstrated that IL-18 is produced mainly by infiltrated macrophages and partly by T lymphocytes and vascular SMCs. In addition, plasma IL-18 levels were dramatically increased in AD blood samples. The plasma IL-18 levels were also positively correlated with the levels of the M1 macrophage-associated cytokines IFN-*γ* and IL-6. Furthermore, the results of a multivariate linear regression analysis showed that IL-18 may be an independent risk factor for diagnosing AD. An anti-IL-18 nAb inhibited M1 macrophage differentiation and SMC apoptosis.

Like most of the other members of the IL-1 superfamily, IL-18 is a powerful proinflammatory cytokine that can participate in many diseases [19]. In an earlier study, Harms et al. reported that circulating IL-18 levels are significantly increased in juvenile T1D patients compared to control subjects and that IL-18 expression is also elevated within pancreatic samples in T1D [20]. A prospective study and an updated meta-analysis found that circulating IL-18 levels are positively associated with coronary heart disease incidence [21]. In addition, circulating and lung IL-18 levels are elevated in PAH patients [22]. Furthermore, an animal experiment found that IL-18 disruption can suppress hypoxia-induced PAH [23]. IL-18 expression has also been reported to be increased approximately 2-fold in human atrial fibrillation patients compared with control subjects and closely related to the incidence of atrial fibrillation [13]. While the IL-18 expression pattern in human AD remains unknown, our present study found that IL-18 levels were increased in both the aorta and plasma of AD patients. These results may suggest that IL-18 is related to the onset of AD.

Previous studies have demonstrated that the immune response and inflammation are closely related to the progression and development of AD. In an earlier study of IL-18, it was reported that granulocyte macrophage colony-stimulating factor (GM-CSF) is a key regulatory and molecular causative agent of aortic dissection/intramural hematoma in a murine model of this condition and is also related to this condition in humans [24]. In addition, T lymphocytes can cause cell death and have been demonstrated to be one of the most important mechanisms underlying the onset of AD [25]. To date, much evidence has suggested that IL-18 can be expressed in dendritic cells, monocytes, and Kupffer cells but especially in macrophages and T lymphocytes [5, 26, 27]. SMCs are the main cell type in the aorta; therefore, double immunofluorescence staining with an anti-IL-18 antibody and an anti-CD68 antibody, anti-*α*-SMA antibody, or anti-CD4 antibody was conducted. We observed that aortic IL-18 expression was predominantly colocalized with macrophages and partially colocalized with T lymphocytes and SMCs in the aorta of AD patients. These results suggested that IL-18 may participate in the occurrence of AD via macrophages.

Data from clinical experiments and animal studies have demonstrated that IL-6 and IFN-*γ* play critical roles in the development and progression of AD. In an earlier study, aortic IL-6 and IFN-*γ* expression was reported to be significantly increased in a mouse AD model [28]. In other studies, Pope et al. and Ju et al. found that high IL-6 levels promoted the progression of AD, while the deletion of IL-6 significantly decreased the onset of AD [29, 30]. Xu et al. reported that circulating IFN-*γ* levels are increased in human AD patients [15]. They also found that the IFN-*γ* level increases in AD patients and is positively correlated with the occurrence of AD [15]. Given that macrophages may be the most important source of IL-6 and IFN-*γ*, to investigate the mechanisms underlying the involvement of IL-18 in AD, we measured circulating IL-6 and IFN-*γ* levels and found that both the IL-6 and IFN-*γ* levels in the plasma were increased and positively associated with the IL-18 level in AD patients. These results suggest that IL-18 may be involved in AD by promoting the secretion of IL-6 and IFN-*γ* by macrophages.

IL-18BP, which is an endogenous antagonist of IL-18, can be induced by IFN-*γ* [31]. To gain a better insight into the biological activity of IL-18 during acute AD, we detected the plasma levels of IL-18BP in the non-AD group and the AD group. We found that high plasma IL-18 levels concurred with increased IL-18BP levels in the AD group. In addition, the calculated levels of free IL-18 were also increased in the acute AD group. IL-18 plays a major role in the production of IFN-*γ*, and IFN-*γ* can stimulate the production of IL-18BP, while IL-18BP can neutralize IL-18 and attenuate the inflammatory response [32]. Therefore, we can presume that IL-18 can promote the production of IFN-*γ* and IL-18BP and overcome endogenous inhibition to promote the occurrence of AD.

Vascular SMCs are an important component of the aortic structure and play a vital role in maintaining the normal structure and function of the aorta in both humans and mice [33]. In addition, to maintain vasoconstriction and diastolic function, SMCs can continuously synthesize and degrade the extracellular matrix (ECM) while maintaining a dynamic balance, which is closely related to the onset and progression of AD because excessive SMC apoptosis can decrease ECM component expression and aortic stability [34, 35]. In fact, an interesting phenomenon was found in the progression of AD; M1 macrophage activity was significantly enhanced, while M2 macrophage activity showed compensatory enhancement but was significantly weaker than the M1 macrophage activity and was not sufficient to counteract the proinflammatory activity of the M1 macrophages [36, 37]. The studies described above demonstrated that in an inflammatory environment, macrophages can convert into proinflammatory M1 macrophages and anti-inflammatory M2 macrophages, which play pathogenic and protective effects, respectively, in the onset and progression of AD, and M1 macrophages play a leading role in the progression of AD. To investigate the mechanisms by which IL-18 participates in the progression of AD, macrophages were treated with Ang II, and the effect of an anti-IL-18 nAb on macrophage differentiation was measured. Because a previous study demonstrated that CD4+ T lymphocytes are essential in the differentiation of macrophages [18], CD4+ T lymphocytes were also added to our cell culture. The results showed that the anti-IL-18 nAb significantly decreased iNOS mRNA expression in macrophages, and similar results for IL-6 and IFN-*γ* were observed after treatment with the anti-IL-18 nAb. The results showed that IL-18 may promote Ang II- and CD4+ T lymphocyte-induced M1 macrophage differentiation, increase M1 macrophage-induced IL-6 and IFN-*γ* expression, and be involved in AD. To further explore the related mechanisms, SMCs were treated with the culture supernatant and Ang II, and the results showed that the anti-IL-18 nAb alleviated M1 macrophage-induced SMC apoptosis. Our results may show that IL-18 promotes M1 macrophage differentiation, increases SMC apoptosis, and accelerates the onset of AD.

In conclusion, IL-18 is widely expressed in various tissues and many cell types and can participate in different immunoregulatory mechanisms. This study is the first to demonstrate a positive association between IL-18 levels and the onset of AD. The possible mechanism is that IL-18 promotes M1 macrophage differentiation and increases SMC apoptosis. Certainly, there are some limitations to our study. First, the small sample size may not have sufficient power to detect a significant relationship between IL-18 and AD. Second, the mechanism of AD is complicated and difficult to clarify, and we explored only the regulatory effect of IL-18 on inflammation. Third, a larger sample size and prospective clinical studies are needed to further verify that IL-18 can contribute to the prognostic assessment of AAD.

## Figures and Tables

**Table 1:** RT-qPCR primers used.

Gene	Forward primer (5′-3′)	Reverse primer (5′-3′)
IL-18 (human)	AAAGATAGCCAGCCTAGAGGTATG	GATCTATCCCCCAATTCATCCT
GAPDH (human)	TTGTCAAGCTCATTTCCTGGT	TTACTCCTTGGAGGCCATGTA
iNOS (mouse)	TGACGCTCGGAACTGTAGCA	CAGTGATGGCCGACCTGAT
IFN-*γ* (mouse)	ACTGGCAAAAGGATGGTGAC	TGAGCTCATTGAATGCTTGG
IL-6 (mouse)	AGTTGCCTTCTTGGGACTGA	TCCACGATTTCCCAGAGAAC
Bax (mouse)	TGAGCGAGTGTCTCCGGCGAAT	GCACTTTAGTGCACAGGGCCTTG
Bcl2 (mouse)	TGGTGGACAACATCGCCCTGTG	GGTCGCATGCTGGGGCCATATA
GAPDH (mouse)	AACTTTGGCATTGTGGAAGG	CACATTGGGGGTAGGAACAC

IL: interleukin; iNOS: inducible NO synthase; IFN: interferon; GAPDH: glyceraldehyde 3-phosphate dehydrogenase.

**Table 2:** Summary of clinical characteristics in patients who provided aortic tissue samples.

Characteristic	NAD	TAD	*P* value
Gender (M/F)	5/2	6/2	0.876
Age (years)	45 (37, 61)	53 (32, 65)	0.315
HBP (*n*, %)	3 (42.8)	6 (75)	0.221
Smoking (*n*, %)	3 (42.8)	5 (62.5)	0.462
Glu (mmol/l)	5.15 (4.83, 5.83)	5.12 (4.83, 5.56)	0.792
SBP (mmHg)	137 (125, 162)	155 (139, 164)	0.254
DBP (mmHg)	90 (78, 111)	82 (78, 96)	0.352
TC (mmol/l)	4.31 (3.95, 4.94)	3.92 (3.74, 4.29)	0.104
TG (mmol/l)	1.21 (0.94, 1.68)	0.92 (0.80, 1.12)	0.102
HDL-C (mmol/l)	1.88 (1.19, 2.14)	1.60 (1.10, 1.92)	0.363
LDL-C (mmol/l)	1.81 (1.52, 3.07)	2.27 (1.28, 2.84)	0.909
CRP (mg/l)	0.95 (0.25, 2.04)	6.06 (4.63, 8.96)	0.021
WBC (×10^9^/l)	5.91 (4.46, 7.86)	14.0 (9.55, 15.15)	0.002
CREA (*μ*mol/l)	68 (58, 74)	74 (59, 97)	0.314
HR (bpm)	59 (57, 78)	78 (70, 85)	0.071
D-dimer (*μ*g/ml)	0.86 (0.69, 0.96)	6.95 (3.26, 9.75)	0.011

HBP: uncontrolled or failure to control of blood pressure; Glu: fasting glucose; SBP: systolic blood pressure; DBP: diastolic blood pressure; TC: total cholesterol; TG: total triglycerides; HDL-C: high-density lipoprotein cholesterol; LDL-C: low-density lipoprotein cholesterol; CRP: C-reactive protein; WBC: white blood cell; CREA: creatinine; HR: heart rate.

**Table 3:** Summary of clinical characteristics in patients who provided blood sample.

Characteristic	NAD	AAD	Stanford A	Stanford B
Gender (M/F)	19/12	70/18^∗^	37/8^∗^	33/10
Age (years)	64 (50, 76)	56 (50, 64)	55 (46, 61)^∗^	61 (53, 69)
Smoking (*n*, %)	6 (19.3)	36 (40.9)^∗^	18 (40)	18 (41.8)^∗^
HBP (*n*, %)	25 (80.6)	79 (89.7)	41 (91.1)	38 (88.3)
Glu (mmol/l)	6.13 (5.6, 6.8)	7.2 (6.4, 8.3)^∗^	7.3 (6.4, 8.2)^∗^	6.8 (6.3, 8.7)^∗^
SBP (mmHg)	156 (146, 170)	145 (128, 167)	144 (121, 161) ^∗^	145 (132, 170)
DBP (mmHg)	84 (76, 93)	80 (70, 94)	75 (66, 92)	80 (74, 97)
WBC (×10^9^/L)	7.5 (4.8, 11.6)	11.0 (8.1, 13.5)^∗^	11.6 (9.5, 14.1)^∗^	10.0 (6.7, 12.9)
TC (mmol/l)	4.0 (3.5, 4.3)	4.07 (3.4, 4.7)	4.13 (3.6, 4.7)	4.0 (3.4, 4.6)
TG (mmol/l)	1.4 (1.2, 1.6)	1.2 (0.9, 1.7)	1.2 (0.9, 1.9)	1.1 (0.9, 1.7)
HDL-C (mmol/l)	1.0 (0.9, 1.1)	1.1 (0.9, 1.4)	1.0 (0.8, 1.3)	1.3 (0.9, 1.5)
LDL-C (mmol/l)	2.1 (1.9, 2.3)	2.2 (1.7, 2.7)	2.3 (1.8, 2.8)	2.0 (1.6, 2.6)
HR (bmp)	72 (63, 84)	78 (68, 90)	78 (60, 84)	80 (72, 91)^∗^
CREA (*μ*mol/l)	72 (62, 92)	82 (68, 106)	80 (67, 94)	85 (75, 113)^∗^
D-dimer (*μ*g/ml)	0.8 (0.6, 1.0)	4.2 (2.0, 6.3)^∗^	4.7 (2.4, 7.2)^∗^	4.0 (1.6, 5.5)^∗^
CRP (mg/l)	18.4 (3.8, 60.9)	10.7 (2.7, 57.4)	11.8 (2.6, 61.3)	9.1 (2.8, 58.8)
Medications, *n* (%)				
Aspirin	5 (16.1)	13 (14.7)	5 (11.1)	8 (18.6)
ACEI/ARB	9 (29.0)	35 (39.7)	14 (31.1)	21 (48.8)
Beta-blockers	4 (12.9)	18 (20.4)	7 (15.5)	11 (25.5)
CCB	9 (29.0)	33 (37.5)	18 (40.0)	15 (34.8)
Diuretics	3 (9.6)	15 (17.0)	6 (13.3)	9 (20.9)

ACEI: angiotensin-converting enzyme inhibitor; ARB: angiotensin receptor blocker; CCB: calcium channel blocker. ^∗^*P* < 0.05 vs. NAD group.

**Table 4:** Plasma cytokines in control and AAD groups.

Cytokines	NAD	AAD	Type A	Type B
IL-18 (pg/ml)	354.1 (293.7, 429.3)	487.3 (406.5, 556.9)^∗^	512.8 (425.6, 560.1)^∗^	479.8 (401.4, 548.8)^∗^
IL-18BP (pg/ml)	821.9 (676.4, 1014.3)	1073 (887, 1260)^∗^	1104 (886, 1275)^∗^	1040 (886, 1140)^∗^
IFN-*γ* (pg/ml)	34.9 (30.8, 40.8)	45.5 (39.9, 52.3)^∗^	46.8 (40.9, 54.7)^∗^	44.3 (39.9, 52.3)^∗^
IL-6 (pg/ml)	355.9 (331.6, 436.1)	475.7 (404.4, 634.3)	503.2 (414.3, 650.9)^∗^	439.3 (399.3, 625.2)^∗^

^∗^ *P* < 0.05 vs. NAD group.

**Table 5:** Association between cytokines, clinical characteristics, and the presence of AAD was assessed by univariate analysis and multivariate linear regression analysis.

Variables	Simple linear			Multiple linear		
	Beta	95% CI	*P* value	Beta	95% CI	*P* value
IL-18	0.509	0.351 to 0.667	0.001	0.238	0.053 to 0.422	0.012
IFN-*γ*	0.522	0.366 to 0.678	0.001	0.232	0.044 to 0.419	0.016
IL-6	0.419	0.252 to 0.585	0.001	0.163	0.002 to 0.324	0.048
Gender	0.185	0.005 to 0.364	0.045	0.124	-0.029 to 0.276	0.111
Smoking	0.198	0.018 to 0.377	0.031	0.137	-0.014 to 0.288	0.075
Glu	0.345	0.173 to 0.517	0.001	0.045	-0.116 to 0.205	0.583
CREA	0.216	0.037 to 0.395	0.018	-0.024	-0.176 to 0.128	0.754
D-dimer	0.464	0.302 to 0.626	0.001	0.179	0.014 to 0.343	0.034
SBP	-0.149	-0.330 to 0.032	0.107			
DBP	-0.102	-0.284 to 0.080	0.269			
TG	-0.068	-0.250 to 0.115	0.465			
TC	0.034	-0.149 to 0.217	0.716			
CRP	-0.028	-0.211 to 0.155	0.760			
WBC	0.140	-0.041 to 0.322	0.128			
Age	-0.130	-0.312 to 0.052	0.159			
HR	0.170	-0.011 to 0.350	0.065			

## Data Availability

The data used to support the findings of this study are available from the corresponding author upon request.
